# Myocardial Damage Patterns in Patients with Left Ventricular Systolic Dysfunction with and Without Coronary Artery Disease Referred for Cardiac Magnetic Resonance †

**DOI:** 10.3390/biomedicines13071612

**Published:** 2025-07-01

**Authors:** Justyna M. Sokolska, Katarzyna Logoń, Magdalena Pszczołowska, Wojciech Kosmala

**Affiliations:** 1 Department of Cardiovascular Imaging, Institute of Heart Diseases, Faculty of Medicine, Wroclaw Medical University, 50-556 Wroclaw, Poland; wojciech.kosmala@umw.edu.pl; 2 Institute of Heart Diseases, Jan Mikulicz-Radecki University Hospital, 50-556 Wroclaw, Poland; 3 Institute of Heart Diseases, Faculty of Medicine, Student Scientific Club of Transplantology and Advanced Therapies of Heart Failure, Wroclaw Medical University, 50-556 Wroclaw, Poland; katarzyna.logon@student.umw.edu.pl (K.L.); magdalena.pszczolowska@gmail.com (M.P.)

**Keywords:** magnetic resonance imaging, late gadolinium enhancement, cardiomyopathy, heart failure, ischemic heart disease

## Abstract

**Background**: Cardiac magnetic resonance (CMR) is widely used to determine the underlying cause of left ventricular (LV) systolic dysfunction. Patients with ischemic disease are less frequently referred for CMR, as the underlying disease is often presumed to explain LV systolic dysfunction. However, various etiologies of myocardial impairment may coexist. Late gadolinium enhancement (LGE) is a technique used for tissue characterization, particularly visualization of myocardial fibrosis. **Objectives**: The aim of this study was to assess the prevalence of LGE patterns suggesting ischemic or non-ischemic etiology of myocardial damage in patients with LV systolic dysfunction with and without known coronary artery disease (CAD). **Methods**: 131 patients (76% male, 55 ± 15 years old) with LV ejection fraction (LVEF) ≤ 50% in echocardiography underwent CMR between December 2021 and November 2022. Patients were divided according to the known history of CAD. Regional subendocardial and transmural LGE was interpreted as ischemic etiology, whereas midmyocardial and subepicardial LGE was non-ischemic. **Results**: The mean LVEF assessed in CMR was 35 ± 10%. A total of 122 patients underwent CMR with LGE sequence. LGE was detected in 62% of patients: 34% had a non-ischemic pattern, 16% ischemic, and 11% mixed. LGE patterns did not differ between patients with and without CAD. In every third patient with CAD and almost every second patient without CAD, no myocardial fibrosis was detected. A completely normal CMR study was found in 6% of patients without CAD and 1% of patients with CAD (all p NS). **Conclusions**: The LGE patterns suggesting ischemic or non-ischemic myocardial damage are similarly prevalent in patients with and without known CAD. The diagnosis based solely on clinical information may be unreliable, as LV dysfunction might have multifactorial origins. The absence of local myocardial fibrosis is relatively common in patients with LV dysfunction, irrespective of its etiology.

## 1. Introduction

Heart failure (HF) is a clinical syndrome that stems from cardiac structural and functional abnormalities leading to impairment in myocardial contractility and/or ventricular filling. HF is one of the biggest challenges in modern medicine. Each year, it is the reason for over 1 million hospitalizations in North America and Europe [1], making HF a huge concern for the healthcare system.

Cardiovascular magnetic resonance (CMR) has become a widely used tool for diagnosing the etiology of HF and determining whether its character is ischemic, non-ischemic, or mixed. CMR imaging provides precise information on tissue characterization, cardiac chamber volumes, and myocardial mass, and allows for the detection of even small local myocardial damage [2,3]. The distribution and extension of late gadolinium enhancement (LGE) obtained with contrast administration can provide etiology-focused data on myocardial injury.

LGE enables the visualization of increased extracellular spaces in conditions such as fibrosis, fibrofatty tissue, or edema. The patterns of LGE help to differentiate between ischemic and non-ischemic myocardial injury. Specific LGE distribution is useful in distinguishing between non-ischemic etiologies of LV systolic dysfunction such as hypertrophic cardiomyopathy, sarcoidosis, myocarditis, amyloidosis, and Fabry disease [4,5].

In recent years, CMR has evolved considerably, with improvements in both imaging sequences and hardware [6]. New methods such as parametric mapping and four-dimensional flow are now being more commonly used in clinical practice. While CMR offers valuable diagnostic information, it also presents certain limitations, particularly long image processing times. Emerging artificial-intelligence-based algorithms have the potential to reduce processing times [7]. Functional CMR has been investigated as a method to evaluate myocardial viability and perfusion. It can assess regional tissue contractility through techniques like MRI tagging, which has been shown to reveal early manifestations of subclinical cardiac dysfunction that typically precede reductions in EF [8].

The purpose of this study was to investigate the consistency of LGE patterns in CMR, suggesting ischemic and non-ischemic etiology of myocardial damage, with the diagnosis of CAD established on the basis of clinical data.

## 2. Materials and Methods

### 2.1. Study Population

This retrospective study was performed in a tertiary cardiology center in Poland. The records of all in- and outpatients who underwent CMR between December 2021 and November 2022 were reviewed. Inclusion criteria were age ≥ 18 years and reduced LV ejection fraction (LVEF ≤ 50%), based on the transthoracic echocardiography, and an unknown HF etiology as the reason for referral for CMR. This article is a revised and expanded version of a paper entitled “The aetiology of myocardial damage in patients with left ventricular systolic dysfunction with and without coronary artery disease referred for cardiac magnetic resonance”, which was presented at the Heart Failure 2023 and the World Congress on Acute Heart Failure, Prague, Czechia, 20–23 May 2023 [9].

After inclusion, information such as demographics, clinical history, comorbidities, and present treatment was collected. For further analysis, the studied population was divided into two groups according to the known history of CAD, including history of ischemic coronary disease diagnosed by the referring cardiologist (based on coronary computed tomography angiography or invasive coronary angiography), past acute coronary syndromes, and revascularization procedures.

The study was conducted in accordance with the Declaration of Helsinki, and authorization to perform this study was obtained from the Wroclaw Medical University Ethical Committee (approval number: 19-285A). All data used for this study were acquired for clinical purposes. Due to the retrospective design of the study, the requirement for written informed consent was waived by the University Ethical Committee. Data processing was performed anonymously and in strict confidence.

### 2.2. Laboratory Measurements

For all inpatients, routine venous blood samples were taken during hospitalization. The following laboratory measurements were performed using standard methods: (i) hematology: hemoglobin, hematocrit, leucocytes, platelets; (ii) renal function tests: creatinine (with a calculation of the estimated glomerular filtration rate (eGFR)); (iii) plasma N-terminal pro-B-type natriuretic peptide (NT-proBNP); (iv) glucose. Outpatients did not have additional venous blood samples taken.

### 2.3. Cardiac Magnetic Resonance Imaging

**Figure A1:**
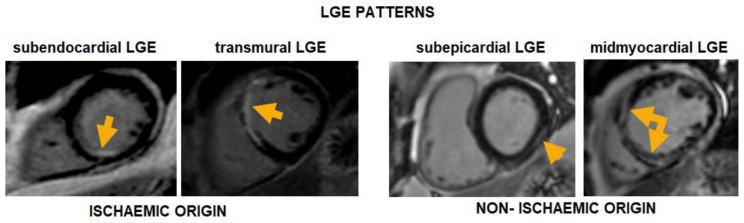
Representative examples of late gadolinium enhancement (LGE) patterns and their interpretation in cardiac magnetic resonance in patients with LVEF ≤ 50% included in the study.

CMR imaging was performed during breath holding at end-expiration using a 1.5 Tesla MRI scanner (Siemens Magnetom Sola Cardiovascular Edition, Siemens Healthineers, Erlangen, Germany) to assess myocardial tissue characteristics and evaluate left and right ventricular diameters, volumes, and function. For all patients, the CMR protocol included cine imaging with a balanced steady-state free precession sequence for cardiac volume assessment (short-axis stack covering the entire left ventricle; two-, three-, and four-chamber views) and LGE imaging to evaluate myocardial scar tissue. LGE imaging was applied in the same orientations as cine images to identify myocardial fibrosis, with Phase-Sensitive Inversion Recovery (PSIR) sequences acquired approximately 10–15 min after the administration of 1.5 mL of gadolinium contrast per kilogram of body weight (gadobutrol, Gadovist 1.0, Bayer Schering Pharma, Zurich, Switzerland). The LGE pattern acquired in CMR with contrast administration was used to determine the possible etiology of myocardial dysfunction in the studied population. Regional subendocardial and transmural LGE was interpreted as of ischemic etiology, whereas midmyocardial and subepicardial LGE was of non-ischemic origin [10] (see Figure A1). CMR images were evaluated using the commercially available CVI42 software (version 5.17, Circle Cardiovascular Imaging, Calgary, AB, Canada).

### 2.4. Statistical Analysis

Statistical analysis was performed using Statistica (StatSoft Polska, version 13.3). Continuous variables were presented as mean with standard deviation (SD). The assumption of normality was tested using the Kolmogorov–Smirnov test. Categorical variables were expressed as counts and percentages. Differences between blood and CMR parameters were assessed by unpaired *t*-tests or respective non-parametric tests if data were not normally distributed. A *p* value equal to or less than 0.05 was considered statistically significant.

## 3. Results

### 3.1. Patient Characteristics

Of 647 CMR studies performed between December 2021 and November 2022, 131 patients (20%) satisfying the inclusion criteria were enrolled. The average age was 55 ± 15 years, 76% were men, the average LVEF assessed by echocardiography was 35%, and 62% of the studied patients had a known CAD history. Previous acute coronary syndrome was diagnosed in 12% of the total studied population, with previous myocardial infarction affecting every tenth person. Regarding co-morbidities, lipid disorders and/or hypertension were present in over half of the studied population, 12% of patients had a history of acute heart failure, and every fifth patient suffered from diabetes. Twenty-three percent of the studied population had at least moderate valve disease, most commonly significant mitral regurgitation (15%) and tricuspid regurgitation (9%). Atrial fibrillation was present in one-third of patients, and 8% had implanted cardiac devices. Among the study participants, 43% were smokers. The most commonly used medications were beta-blockers, SGLT-2 inhibitors, aldosterone antagonists, and statins. Detailed baseline characteristics of the study population are presented in Table 1.

### 3.2. Studied Subgroups

The studied group was divided into 2 subgroups: patients with and without known CAD in their medical history, accounting for 62% and 38% of the population, respectively.

Among comorbidities, significant differences between studied subgroups were observed in liver diseases and asthma, which were more common in the group without CAD. In this group, aortic regurgitation in a moderate/severe state was observed significantly more often than in those with CAD (9 vs. 1%, *p* = 0.04).

In 117 patients, venous blood samples were taken. Regarding the laboratory findings, the number of blood platelets was significantly higher in the group with CAD.

There were no differences between the two groups in terms of medications used, except P2Y12 inhibitors, which were used by 16% of the patients with CAD and no patients without known CAD. Furthermore, 20% of the CAD subgroup had previous acute coronary syndromes, including myocardial infarction with/without ST elevation in 16% of this subgroup. Detailed baseline characteristics of the study subgroups are presented in Table 1.

### 3.3. CMR Findings

A total of 122 (93%) patients underwent CMR study with contrast administration and were included into further analysis of LGE presence and patterns, whereas in 9 patients, contrast was not administered due to chronic kidney disease/dialysis (n = 5), or because the study was interrupted as there were non-diagnostic cine images with severe artifacts coming from implanted devices (ICD n = 3, CRT-D n = 1).

Patients with CAD had lower LVEF, more dilated left ventricles, and higher LV mass than those without CAD. There were no relevant differences in the right ventricle between the studied subgroups (Table 2).

**Figure 1:**
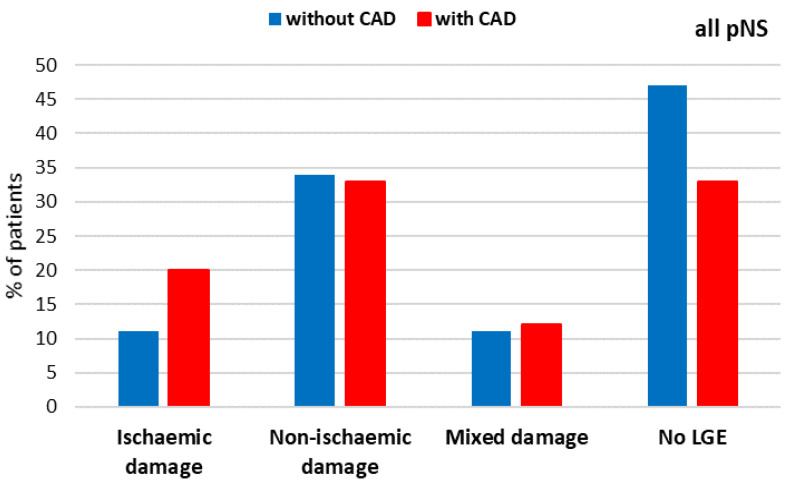
Distribution of myocardial damage based on LGE interpretation in patients with LVEF ≤ 50%.

CMR revealed that LGE was present in the majority of the studied HF population. Subepicardial and/or midmyocardial LGE, suggesting non-ischemic myocardial damage, was found in half of the studied group, whereas subendocardial LGE, indicating ischemic myocardial damage, was found in almost every third patient. Every tenth patient had both patterns of myocardial damage (Table 3, Figure 1).

**Figure 2:**
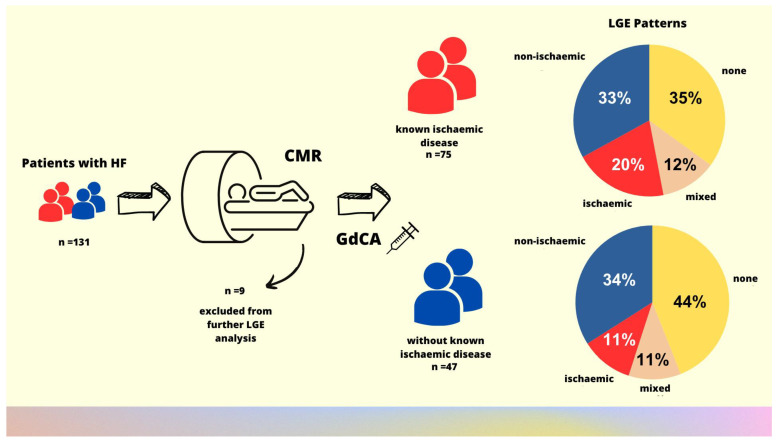
Graphical abstract. Study flowchart and the main study findings showing the distribution of etiology of myocardial damage based on LGE patterns in CMR in patients with heart failure.

There were no differences between subgroups with and without known CAD in the frequency of LGE patterns. However, patients with known CAD had, on average, twice as many cardiac segments with subendocardial scarring as those without a previous diagnosis of CAD. The number of segments with midmyocardial LGE was significantly higher among subjects without CAD (Table 3). Among patients with a history of acute coronary syndromes and/or revascularization procedures, the most common LGE pattern was ischemic, observed in 44% of cases. A mixed pattern was present in 22% of patients, while a non-ischemic pattern was present in 17%. In one patient, no myocardial fibrosis was detected. A summary of CMR findings, together with a study flowchart, is presented in Figure 2.

## 4. Discussion

This study uniquely demonstrates that the CMR-derived LGE patterns, typically used to distinguish ischemic from non-ischemic etiologies of myocardial injury, are equally prevalent in patients with and without known CAD. This finding highlights the limitations of relying solely on clinical history or LGE distribution in determining the etiology of LV systolic dysfunction, underscoring the multifactorial nature of heart failure and advocating for a more comprehensive diagnostic approach.

The study showed that myocardial fibrosis may not correspond to the previous history of CAD. The most common type of myocardial damage was non-ischemic, which was present in every third studied patient. Ischemic pattern was observed less often, whereas coexistence of both etiologies was found only in every ninth patient. Importantly, no differences in LGE patterns were found between patients with and without known CAD. The presence of both ischemic and non-ischemic LGE among patients with and without known CAD suggests the contribution to LV impairment from different etiological factors. This highlights the importance of considering a range of reasons for cardiac impairment in clinical decision making, irrespective of past medical information.

In the cohort of patients with HF, two-thirds were previously diagnosed with coronary artery disease (CAD), which is consistent with previous studies, demonstrating CAD to be the most common etiological factor in HF [11,12,13]. In patients with known CAD, only one-fifth had isolated ischemic scars, whereas many patients had non-ischemic patterns of myocardial damage, suggesting the presence of mixed etiology. It is an important finding as non-ischemic fibrosis was found to be associated with a poorer outcome than fibrosis attributable to myocardial infarction [14]. In all-cause mortality analysis, both ischemic and non-ischemic LGE patterns were associated with worse survival than the reference group. However, in the adjusted analysis, only major non-ischemic LGE (defined as well-established, classic patterns associated with myocarditis, infiltrative cardiomyopathies, or myocardial hypertrophy) was associated with an elevated risk of mortality [14].

There was no significant difference in LGE patterns between patients with and without known CAD. Interestingly, in patients without a history of CAD, LGE patterns associated with ischemic scars are found as frequently as in patients with known CAD. Every fifth patient without CAD presented with a subendocardial LGE pattern, associated with ischemic scars. This finding suggests a possible ischemic etiology of HF with previously undetected myocardial infarction in these subjects. Results from this study are consistent with a previous report [15].

In a large proportion (39%) of the currently studied population, no imaging findings of replacement myocardial fibrosis were detected. However, only a few patients had no other pathologies detected in CMR.

### 4.1. Clinical Implications

This study provides evidence that patient management strategy should not focus solely on previous medical findings and highlights that LV dysfunction might have multifactorial origins, even in the presence of established CAD. Comprehensive management of all underlying conditions is essential for achieving better clinical outcomes.

The pattern and extent of myocardial fibrosis predict adverse outcomes in new-onset HFrEF [16], the development of LV dysfunction [17], and the need for heart transplantation, regardless of the presence of CAD [18]. Therefore, the detection of fibrosis by CMR can provide additional prognostic information and improve risk stratification. This is crucial in view of a recent meta-analysis demonstrating the prognostic importance of unrecognized myocardial infarctions [19,20].

### 4.2. Limitations of the Study

This study has several limitations that should be acknowledged. First, the possible presence of asymptomatic CAD was not excluded by computed tomography or coronary angiography in all patients from the non-CAD group. Second, the retrospective design and cross-sectional character of the study inherently limit the ability to establish causality. Third, the single-center study design constrains the generality of our findings, as variations in clinical practice, patient demographics, and local healthcare systems could influence the outcomes. Fourth, the relatively small sample size may have contributed to the limited ability to detect certain intergroup differences, particularly in the frequency of the isolated ischemic LGE pattern. Fifth, while the analysis of the underlying etiology was based on widely accepted interpretations of LGE patterns, this approach may have introduced interpretative bias, particularly if subtle variations in LGE patterns were not adequately captured or if there were inconsistencies in the imaging protocol. Sixth, the reliance on imaging findings without histopathologic confirmation further limits the reliability of conclusions, as LGE patterns, although informative, are not definitive indicators of specific pathologies. The lack of histopathologic correlation means that some diagnoses might have been inaccurately classified, potentially affecting the validity of the results. Lastly, incomplete or missing information for some patients might have impacted the robustness of our findings.

Future research should aim to address these limitations by utilizing a prospective, multicenter design, incorporating histopathologic validation, and including a more comprehensive analysis of potential confounding factors to strengthen the validity and generalizability of findings.

## 5. Conclusions

LGE patterns suggesting ischemic or nonischemic etiology of LV systolic dysfunction are similarly prevalent in patients with and without a known history of CAD. Therefore, establishing the diagnosis of LV damage based on clinical information alone may be unreliable. The ischemic LGE patterns in patients without known CAD suggest the possibility of undiagnosed myocardial infarctions contributing to LV dysfunction. Non-ischemic or mixed LGE patterns in patients with CAD indicate additional contributing factors of LV dysfunction besides CAD. The absence of replacement myocardial fibrosis is a quite common finding in patients with LV dysfunction, irrespective of its etiology.

## Figures and Tables

**Table 1:** Baseline characteristics of the study population and studied subgroups.

Parameters	Studied Population n = 131	Patients Without CAD n = 50 (38%)	Patients with CAD n = 81 (62%)	*p*
Demographics				
Age (years)	55 ± 15	55 ± 16	55 ± 15	0.90
Male sex, n (%)	100 (76)	38 (76)	62 (77)	0.94
LVEF, % (echocardiography)	35 ± 10	39 ± 9	33 ± 10	0.006 *
Clinical data				
Known coronary artery disease (CAD), n (%); including previous acute coronary syndromes (UA and/or MI), n (n%)	81 (62); 18 (14)	0 0	81 (100) 18 (22)	<0.001 *
Hypertension, n (%)	66 (54)	21 (48)	45 (57)	0.32
Atrial fibrillation, n (%)	42 (34)	17 (39)	25 (32)	0.43
Significant (moderate or severe) valve disease, n (%)	28 (23)	9 (20)	19 (24)	0.58
Chronic kidney disease, n (%)	23 (19)	8 (18)	15 (19)	0.91
Liver diseases, n (%)	13 (11)	6 (14)	7 (9)	0.04 *
Chronic obstructive pulmonary disease/asthma, n (%)	4 (3)	4 (9)	0	0.02 *
Number of comorbidities, n (%)	2.9 ± 1.8	1.9 ± 1.3	3.4 ± 1.8	<0.001 *
Basic laboratory parameters				
Hemoglobin (g/dL)	14.4 ± 2.0	14.6 ± 1.9	14.2 ± 2.0	0.69
Platelets (10^3^/μL)	225 ± 69	203 ± 53	237 ± 74	0.01 *
Creatinine (mg/dL)	1.24 ± 1.22	1.15 ± 1	1 ± 1	0.55
NTproBNP (pg/L)	3433 ± 8131	2639 ± 5683	3782 ± 9011	0.51
Treatment				
Loop diuretics, n (%)	66 (57)	19 (45)	47 (64)	0.06
ACEI/ARB, n (%)	43 (37)	17 (41)	26 (35)	0.53
Sacubitril + valsartan, n (%)	44 (38)	17 (41)	27 (36)	0.63
Beta-blockers, n (%)	100 (86)	38 (90)	62 (83)	0.25
Aldosterone antagonists, n (%)	81 (69)	28 (67)	53 (71)	0.65
SGLT-2 inhibitors, n (%)	78 (67)	27 (64)	51 (68)	0.68
Statins, n (%)	69 (59)	25 (60)	44 (59)	0.93
Acetylsalicylic acid, n (%)	27 (23)	6 (14)	21 (28)	0.09
P2Y12 inhibitors (clopidogrel/prasugrel/ticagrelor), n (%)	12 (10)	0	12 (16)	0.006 *
Dual antiplatelet therapy, n (%)	9 (7)	0	9 (11)	0.003 *

Results are presented as numbers with a percentage or an average number with standard deviation. Abbreviations: ACEI, angiotensin-converting enzyme inhibitors; ARB, angiotensin II receptor blockers; CABG, coronary artery bypass grafting; CAD, coronary artery disease; LVEF, left ventricular ejection fraction; MI, myocardial infarction; * A *p* value of 0.05 or lower was considered significant.

**Table 2:** Cardiac magnetic resonance characteristics in the studied population.

Parameters	Studied Population n = 131	Patients Without CAD n = 50	Patients with CAD n = 81	*p*
Left ventricle and atrium				
IVSd (mm)	10 ± 2	10 ± 2	10 ± 2	0.87
LVEDd (mm)	65 ± 9	62 ± 8	67 ± 9	0.004 *
LVESd (mm)	52 ± 12	49 ± 9	55 ± 12	0.01 *
LVEDVi (mL/m^2^)	124 ± 37	115 ± 28	130 ± 42	0.04 *
LVESVi (mL/m^2^)	80 ± 37	70 ± 27	88 ± 42	0.01 *
LVEF (%)	38 ± 12	41 ± 11	35 ± 12	0.004 *
LV-CO (mL/min)	6 ± 6	7 ± 5	6 ± 6	0.54
LVMassEDi (g/m^2^)	72 ± 19	69 ± 17	73 ± 20	0.2
LAVI (mL/m^2^)	50 ± 19	50 ± 2	50 ± 19	0.88
Right ventricle and atrium				
RVEDVi (mL/m^2^)	89 ± 27	89 ± 22	89 ± 30	0.96
RVESVi (mL/m^2^)	47 ± 26	48 ± 25	46 ± 27	0.74
RVEF (%)	50 ± 11	49 ± 10	50 ± 12	0.88
RAVI (mL/m^2^)	44 ± 19	47 ± 21	42 ± 18	0.18

Abbreviations: CAD, coronary artery disease; IVSd, interventricular septum end-diastolic diameter; LAVI, left atrial volume index; LV-CO, left ventricular cardiac output; LVEDd, left ventricular end-diastolic diameter; LVEDVi, left ventricular end-diastolic volume index; LVEF, left ventricular ejection fraction; LVESd, left ventricular end-systolic diameter; LVESVi, left ventricular end-systolic volume index; LVMassEDi, left ventricular end-diastolic mass index; RAVI, right atrial volume index; RVEDVi, right ventricular end-diastolic volume index; RVEF, right ventricular ejection fraction. * A *p* value of 0.05 or lower was considered significant.

**Table 3:** Cardiac magnetic resonance—late gadolinium enhancement characteristics in the studied population.

Parameters	Studied Population n = 131	Patients Without CAD n = 50	Patients with CAD n = 81	*p*
LGE sequence, n (%)	122 (93)	47 (94)	75 (93)	0.76
Presence of LGE, n (%)	75 (61)	26 (55)	49 (65)	0.14
Subendocardial and transmural pattern of LGE, n (%)	34 (28)	10 (21)	24 (32)	0.20
Cardiac segments with subendocardial and transmural LGE, n	1.0 ± 2.1	0.6 ± 1.3	1.2 ± 2.5	<0.001 *
Subepicardial pattern of LGE, n (%)	9 (7)	5 (11)	4 (5)	0.28
Cardiac segments with subepicardial LGE, n	0.3 ± 1.3	0.4 ± 1.3	0.3 ± 1.3	0.92
Midmyocardial pattern of LGE, n (%)	52 (43)	19 (40)	36 (44)	0.70
Cardiac segments with midmyocardial LGE, n	1.4 ± 2.3	1.5 ± 2.9	1.3 ± 1.8	<0.001 *
Subepicardial and midmyocardial pattern of LGE, n (%)	55 (45)	21 (45)	34 (45)	0.94
Interpretation of myocardial damage				
Isolated ischemic myocardial damage, n (%)	20 (16)	5 (11)	15 (20)	0.17
Isolated non-ischemic myocardial damage, n (%)	41 (34)	16 (34)	25 (33)	0.94
Mixed myocardial damage, n (%)	14 (11)	5 (11)	9 (12)	0.82
Normal CMR findings (LVEF ≧ 51% in men and >52% in women)	4 (3)	3 (6)	1 (1)	0.12

Abbreviations: CMR, cardiovascular magnetic resonance; LGE, late gadolinium enhancement; LVEF, left ventricular ejection fraction; * A *p* value of 0.05 or lower was considered significant.

## Data Availability

The data underlying this article will be shared upon reasonable request to the corresponding author.

## Abbreviations

The following abbreviations are used in this manuscript:

CMR	Cardiac magnetic resonance
LV	Left ventricle
CAD	Coronary artery disease
LVEF	Left ventricular ejection fraction
LGE	Late gadolinium enhancement
HF	Heart failure
PSIR	Phase-sensitive inversion recovery
